# miR-503-5p confers drug resistance by targeting PUMA in colorectal carcinoma

**DOI:** 10.18632/oncotarget.15559

**Published:** 2017-02-21

**Authors:** Ke Xu, Guo Chen, Yanyan Qiu, Zeting Yuan, Hongchang Li, Xia Yuan, Jian Sun, Jianhua Xu, Xin Liang, Peihao Yin

**Affiliations:** ^1^ Central Laboratory, Putuo Hospital, Shanghai University of Traditional Chinese Medicine, Shanghai 200062, PR China; ^2^ Interventional Cancer Institute of Chinese Integrative Medicine, Shanghai University of Traditional Medicine, Shanghai 200062, PR China; ^3^ Department of Radiation Oncology, School of Medicine and Winship Cancer Institute of Emory University, Atlanta, Georgia 30322, USA; ^4^ Department of General Surgery, Putuo Hospital, Shanghai University of Traditional Chinese Medicine, Shanghai 200062, PR China State; ^5^ Department of Pharmacy, Putuo Hospital, Shanghai University of Traditional Chinese Medicine, Shanghai 200062, PR China; ^6^ State Key Laboratory of Bioreactor Engineering & Shanghai Key Laboratory of New Drug Design, School of Pharmacy, East China University of Science and Technology, Shanghai 200237, PR China

**Keywords:** colorectal carcinoma, multidrug-resistance, miR-503-5p, PUMA, p53

## Abstract

The development of multidrug-resistance (MDR) is a major contributor to death in colorectal carcinoma (CRC). Here, we investigated the possible role of microRNA (miR)-503-5p in drug resistant CRC cells. Unbiased microRNA array screening revealed that miR-503-5p is up-regulated in two oxaliplatin (OXA)-resistant CRC cell lines. Overexpression of miR-503-5p conferred resistance to OXA-induced apoptosis and inhibition of tumor growth *in vitro* and *in vivo* through down-regulation of PUMA expression. miR-503-5p knockdown sensitized chemoresistant CRC cells to OXA. Our studies indicated that p53 suppresses miR-503-5p expression and that deletion of p53 upregulates miR-503-5p expression. Inhibition of miR-503-5p in p53 null cells increased their sensitivity to OXA treatment. Importantly, analysis of patient samples showed that expression of miR-503-5p negatively correlates with PUMA in CRC. These results indicate that a p53/miR-503-5p/PUMA signaling axis regulates the CRC response to chemotherapy, and suggest that miR-503-5p plays an important role in the development of MDR in CRC by modulating PUMA expression.

## INTRODUCTION

Colorectal carcinoma (CRC) is the third leading cause of tumor-related death in the world [1], and it is also one of the most shared malignant tumors in China. Oxaliplatin is the third-generation platinum compound, which is the first one to show efficacy in the colorectal carcinoma treatment of platinum-based compounds [2]. Oxaliplatin induces formation of intra-strand guanine-guanine and guanine-adenine DNA links, cell cycle arrest, and death of proliferating cells [3]. Resistance to oxaliplatin is multifactorial, and includes low-efficient cellular drug uptake and accumulation [4], activation of the antioxidant glutathione system for detoxification [5, 6], enhancement of DNA repair [7], and up-regulation of anti-apoptosis pathways [8–11]. In order to comprehend the molecular mechanisms of CRC oxaliplatin resistance, our laboratory has established an *in vitro* chemoresistant CRC cell line model by chronic exposure of human CRC cells (HT29 & HCT116) to increasing doses of oxaliplatin.

MicroRNAs (miRNA) are single-stranded non-coding RNAs, which could silent gene by binding to the three prime untranslated regions (3′ UTRs) complementary sequences of the target messenger RNA transcripts (mRNAs)[12, 13]. MiRNAs only account about 1% of all human genes, but they are predicted to adjust up to 30% of human protein-coding genes expression [14–18]. Aberrant miRNA expression has been reported in several types of malignancies, including CRC [19–21]. However, the mechanisms of miRNA involvement in the acquired drug resistance of CRC cells are largely unknown. Our previous studies have suggested that downregulation of miRNAs may modulate drug resistance in colorectal carcinoma by targeting multidrug resistance (MDR) proteins [22–25].

The p53 up-regulated modulator of apoptosis (PUMA) is a BH3 domain only pro-apoptotic protein belonging to the Bcl-2 family, also known as BBC3 (Bcl2 binding component 3). PUMA is an direct downstream target of p53, but it still could induce p53-independent apoptosis to a variety of stimulus [26–29]. p53 could be altered in more than 50% of human cancers as a tumor suppressor gene, which plays crucial roles in apoptosis, DNA repair or cell cycle arrest [30–32]. And miRNA expression could also be regulated by p53 in both transcription-dependent (e.g. miR-34) and transcription-independent way (e.g. miR-15, miR-143, and miR-1915) [33, 34].

In this examine, we have found a novel p53/miR-503-5p/PUMA signaling way that regulates the response of colorectal carcinoma cells to oxaliplatin. We demonstrate that p53 suppresses expression of miR-503-5p and miR-503-5p could increase after p53 deletion. Inhibiting miR-503-5p expression in p53 Knock-out cells up-regulate the their sensitivity to oxaliplatin. miR-503-5p induces oxaliplatin resistance through the inhibition of apoptosis by reducing PUMA expression, which could direct target by miR-503-5p. In addition, a CRC xenograft mouse model be using manifest that miR-503-5p reduce the effect of oxaliplatin to CRC *in vivo* and inhibition of miR-503-5p increase oxaliplatin sensitive to CRC drug resistance cells *in vivo*. Importantly, examine of colorectal carcinoma tissues evidence a negative correlation between miR-530-5p and PUMA expression. Together, this study has identified that miR-503-5p could play a crucial role in the drug resistance of CRC by modulating PUMA expression, and it will be a potential MDR treatment target in CRC.

## RESULTS

### miR-503-5p is overexpressed in oxaliplatin-resistant CRC cells

**Figure 1:**
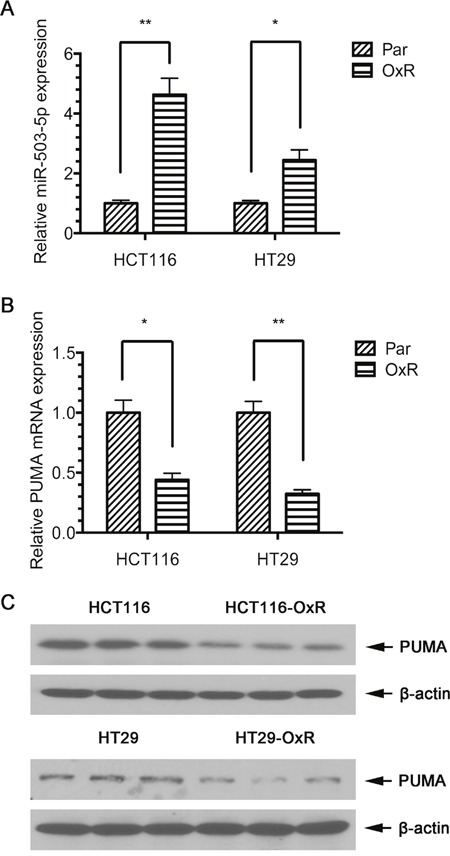
Expression of miR-503-5p and PUMA in human CRC cell lines **A**. qPCR validation of miR-503-5p expression levels significantly increased in oxaliplatin-resistant (OXR) CRC cells. **B**. qPCR validation of PUMA mRNA expression levels significantly decreased in oxaliplatin-resistant (OXR) CRC cells. **C**. Western blot validation of PUMA protein expression levels significantly decreased in oxaliplatin-resistant (OXR) CRC cells. Abundance of miR-503-5p and PUMA was normalized to U6 RNA, GAPDH and β-actin, respectively. Results are representative of three experiments.

In order to define whether microRNAs could be involved in the development of drug-resistance in CRC, we compared the miRNA expression profiles of CRC cell and their oxaliplatin-resistant cell (HCT116 and HT29) by using miRNA microarray (including 2578 human mature miRNA). Table 1 lists 10 miRNAs that were differentially expressed between the parental cells and the drug-resistant cells. Since miR-503-5p expression was increased in both oxaliplatin-resistant cell lines, we hypothesized that miR-503-5p plays a paramount role in acquired oxaliplatin-resistant CRC cells. Furthermore, to confirm the results obtained by microarray profiling, we used RealtimePCR to analysis miR-503-5p expression in the above 4 cell lines. And the results manifested miR-503-5p up-regulated in the HCT116-OxR and HT29-OxR cells as same as the microarray data (Figure 1A).

**Table 1:** miRNAs differentially expressed in HCT116, HT29 and their oxaliplatin resistance cells

miRNA	HCT116 OXR/PAR	HT29 OXR/PAR
hsa-miR-203	1.82	3.12
has-miR-222	0.332	0.412
has-miR-297	0.254	0.521
hsa-miR-338-5p	0.401	0.308
***hsa-miR-503-5p***	***3.98***	***2.02***
hsa-miR-630	0.302	0.421
hsa-miR-939	2.12	1.85
hsa-miR-1202	2.95	1.58
hsa-miR-1258	0.426	0.481
hsa-miR-1915	0.281	0.392

To determine miR-503-5p target genes, three algorithms be used to predict the target gene of miRNAs – miRBase (http://www.mirbase.org), PicTar (http://pictar.mdc-berlin.de/), and TargetScan (http://www.targetscan.org). Based on the representation of miR-503-5p recognition sites in their 3′ UTRs, we found that one of the candidate target genes, PUMA, showed reduced expression of mRNA and protein levels in HCT116-OxR and HT29-OxR cells compared to their parental cells (Figure 1B & 1C), suggesting that miR-503-5p might induce oxaliplatin resistance in CRC cells by targeting PUMA.

### PUMA is a posttranscriptional repression target of miR-503-5p

**Figure 2:**
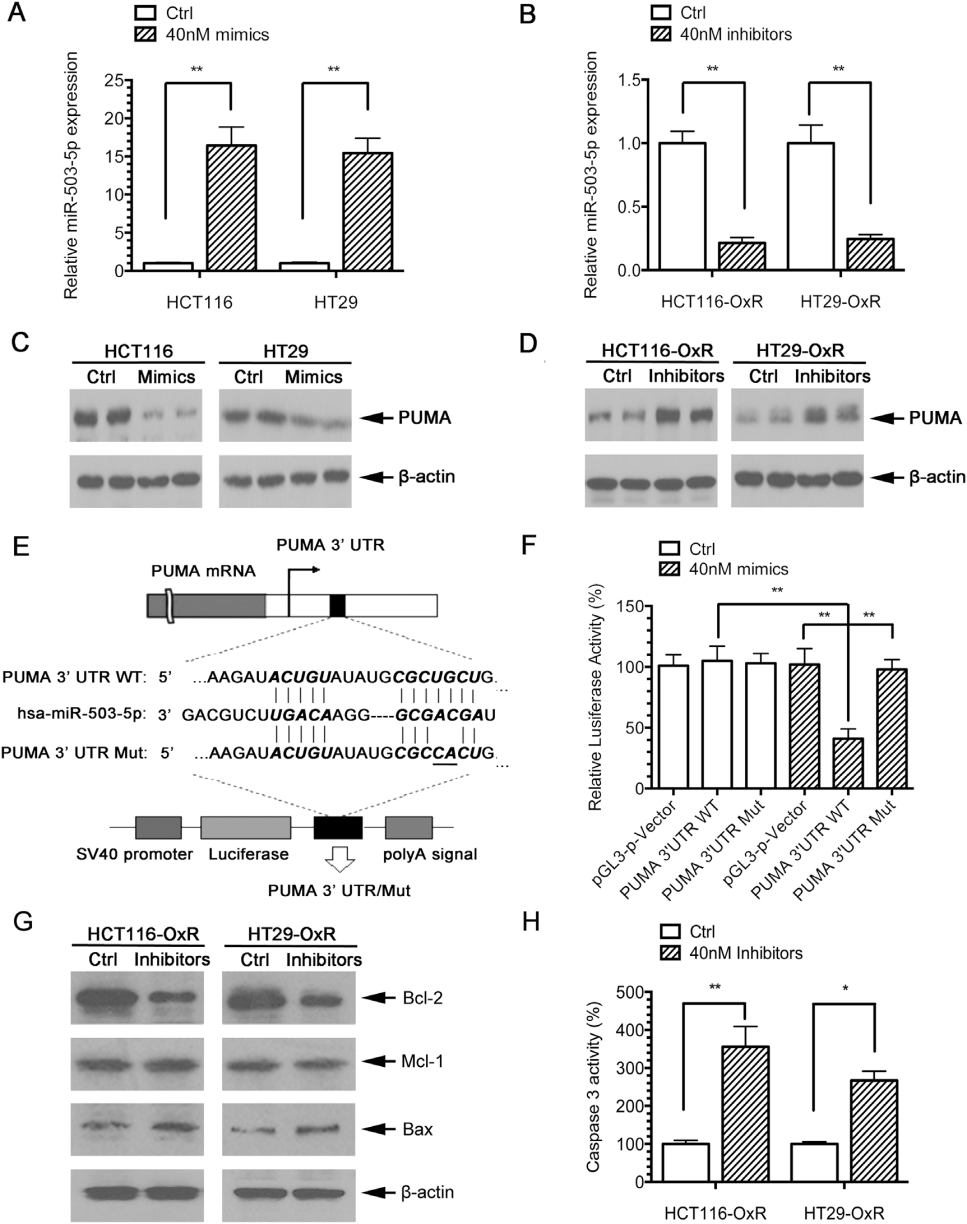
PUMA is a direct target of miR-503-5p **A**. MiR-503-5p expression level was significantly increased after transient transfection of miR-503-5p mimic in CRC parental cells as measured by qPCR. **B**. MiR-503-5p expression level was significantly decreased after transient transfection of miR-503-5p inhibitor in in CRC oxaliplatin-resistant cells as measured by qPCR **C**. PUMA protein expression levels are regulated directly by miR-503-5p, as reflected by decreased PUMA expression in CRC-Par cells after transient transfection of miR-503-5p mimic **D**. and increased PUMA expression in CRC-OxR cells after miR-503-5p inhibitor transfection. **E**. The wild-type and mutant variant of the putative miR-503-5p target sequences of the PUMA gene. TargetScan predicts one binding sites in the PUMA 3′UTR. **F**. Two copies of the wild-type and mutant miR-503-5p target sequences were fused with a luciferase reporter and transfected into control oligonucleotide and 40 nM miR-503-5p mimics infected HCT116-OxR cells. MiR-503-5p significantly suppressed the luciferase activity of the wild-type PUMA 3′UTR. **G**. Bcl-2, Mcl-1 and Bax protein levels were assessed 48 h after transfection of control oligonucleotide and MiR-503-5p inhibitors (40 nM) HCT116-OxR cells, which were respectively detected by Western blotting. **H**. The percentage of caspases-3 activity was assessed in HCT116-OxR cells as described above. **p<0.01. Results are representative of three experiments.

The increased expression of miR-503-5p in CRC-OxR cells was associated with the decreased protein expression of PUMA. To determine the influence of miR-503-5p on the PUMA expression, we transfected HCT116 and HCT29 cells with miR-503-5p mimics or control mimics, and transfected HCT116-OxR cells and HCT29-OxR cells with miR-503-5p inhibitors or control inhibitors. The qPCR results showed that the miR-503-5p mimics increased miR-503-5p expression levels in HCT116 and HT29 cells (Figure 2A), while miR-503-5p inhibitors decreased the expression (Figure 2B). The PUMA protein levels were inhibited in HCT116 and HCT29 cells after miR-503-5p mimic-transfected (Figure 2C), and increased in HCT116-OxR and HCT29-OxR cells after miR-503-5p inhibitor-transfected (Figure 2D).

To determine whether miR-503-5p directly targets PUMA, a section of PUMA 3′ UTR including the miR-503-5p binding site was cloned into a luciferase reporter system, and a miR-503-5p binding site lacking plasmid was used as a negative control for the system(Figure 2E). Both the reporter plasmid and miR-503-5p mimics were transfected into the HCT116-OxR cells. The results demonstrated that luciferase activity of PUMA 3′ UTR fragment containing the miR-503-5p binding site is inhibited by miR-503-5p. And no effect was observed when the plasmid without the miR-503-5p binding sites (Figure 2F).

To examine whether miR-503-5p also regulates other apoptotic proteins, expression of Bcl-2, Bax, and Mcl-1 was analyzed by western blotting in miR-503-5p inhibitors transfected HCT116-OxR cells. Figure 2G shows that miR-503-5p inhibitors also modulate those above proteins. As PUMA is a post-transcriptional repression target of miR-503-5p, we also tested whether miR-503-5p inhibitors could regulate caspase activity. Figure 2H shows that miR-503-5p inhibitors activate caspase-3 in the drug resistant cells. Taken together, these data indicate that miR-503-5p decreases expression of PUMA by targeting its 3′ UTR directly, and miR-503-5p could also modulates apoptosis level by PUMA.

### miR-503-5p induces oxaliplatin resistance in CRC cells

**Figure 3:**
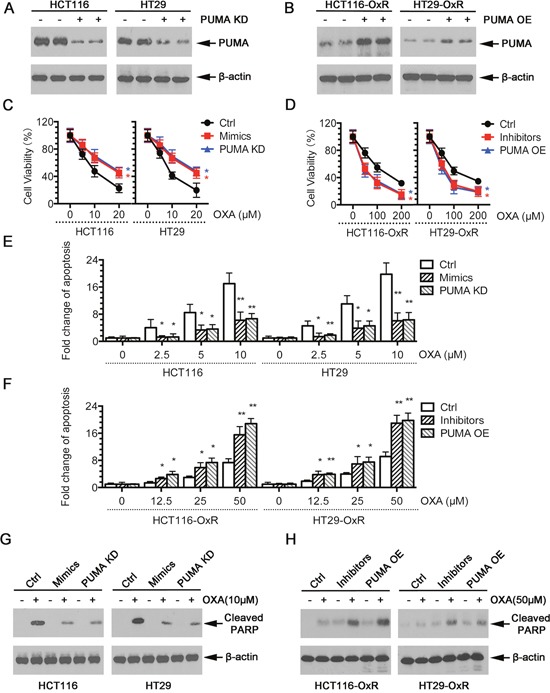
Modulation of miR-503-5p expression altered the sensitivity of CRC cells to oxaliplatin *in vitro* **A**. Expression of PUMA was knocked down by shRNAs in CRC-Par cells. **B**. Expression of PUMA was overexpression by Plasmids in CRC-OxR cells. **C**. Overexpression of miR-503-5p induced by mimics transfection significantly decreased the growth-inhibitory effect of oxaliplatin in CRC-Par cells by MTT assay; **E**. decreased CRC-Par cells apoptosis level to oxaliplatin by DNA fragmentation assays; **G**. and decreased cleaved PARP level by western blot analysis, same effect as PUMA knock down (PUMA KD). **D**. Down-regulation of miR-503-5p induced by inhibitors transfection significantly slowed down CRC-OxR cells growth under oxaliplatin treatment by MTT assay; **F**. increased CRC-OxR cells apoptosis level to oxaliplatin by DNA fragmentation assays; **H**. and increased cleaved PARP level by western blot analysis, same effect as PUMA overexpression (PUMA OE). *p<0.05, **p<0.01. Results are representative of three experiments.

To confirm the functional role of miR-503-5p in inducing chemo-resistance by PUMA, we used PUMA overexpression (PUMA OE) plasmid and PUMA knock down (PUMA KD) shRNA plasmid (Figure 3A). The results showed that increased level of miR-503-5p and decreased level of PUMA protected the oxaliplatin-induced apoptosis in HCT116 and HT29 cells, as evidenced by a right shift of the growth inhibition curve (Figure 3C), significantly reduced apoptosis (Figure 3E) and decreased levels of cleaved PARP (Figure 3G). Conversely, inhibition of miR-503-5p and overexpression of PUMA re-sensitized HCT116-OxR and HT29-OxR cells to oxaliplatin treatment, as evidenced by a left shift of the growth inhibition curve (Figure 3D), increased apoptosis (Figure 3F), and increased cleavage of PARP (Figure 3H). The above data demonstrate that miR-503-5p could modulate the oxaliplatin-resistance phenotype of CRC cells.

### miR-503-5p reduces effectiveness of oxaliplatin in CRC *in vivo*

**Figure 4:**
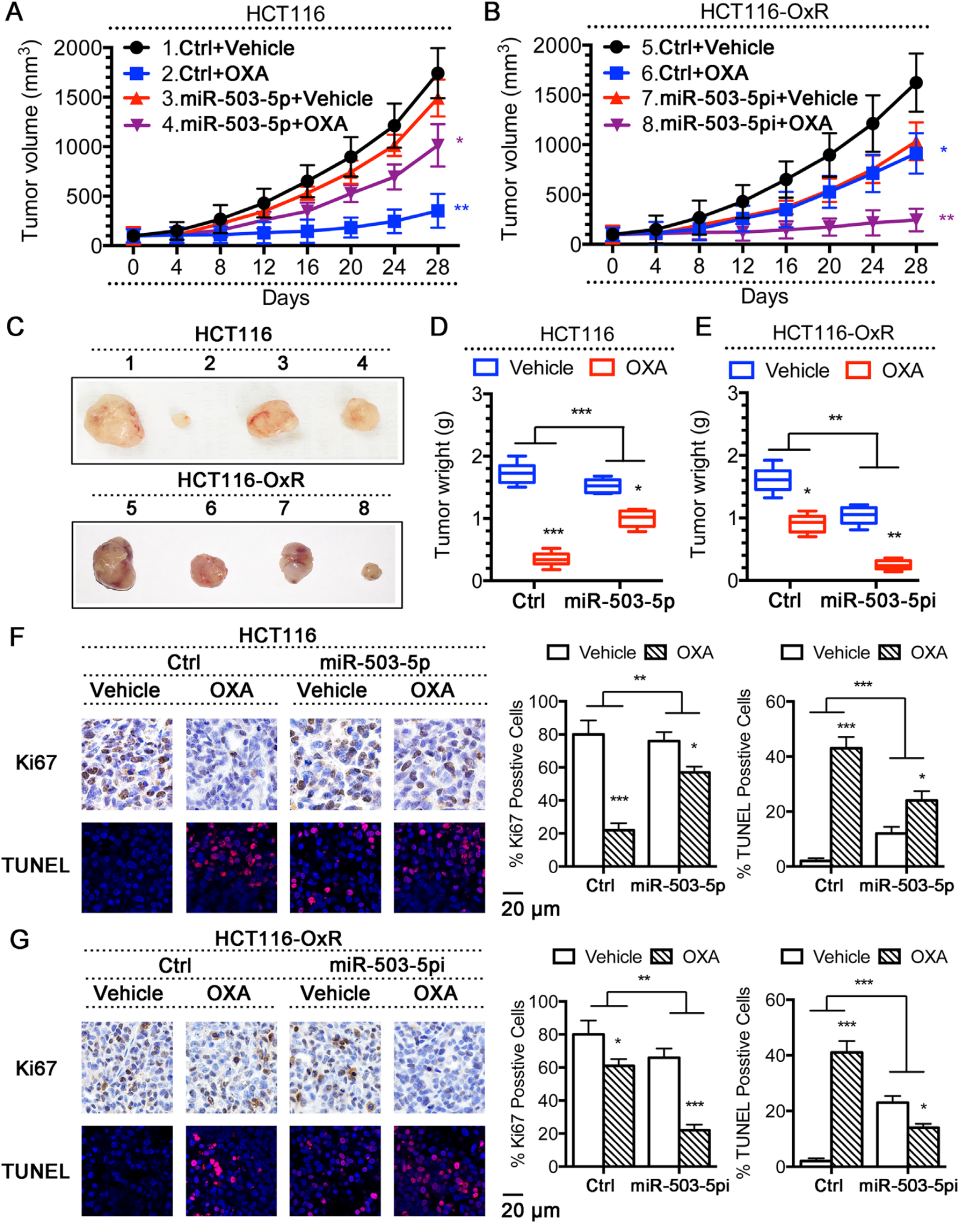
Modulation of miR-503-5p expression altered the sensitivity of CRC cells to oxaliplatin *in vivo* Overexpression of miR-503-5p reduced the effectiveness of OXA in the inhibition of tumor growth *in vivo*. **A**. Xenograft tumor growth curves, **C. (up panel)** pictures of tumors taken on the same scale **D**. and tumor weights. Down-regulation of miR-503-5p increased the effectiveness of OXA in the inhibition of tumor growth *in vivo*. **B**. Xenograft tumor growth curves, **C. (down panel)** pictures of tumors taken on the same scale **E**. and tumor weights. **F**. Overexpression of miR-503-5p significantly decreased the growth-inhibitory effect of oxaliplatin in CRC-Par cells by Ki67 level, and decreased CRC-Par cells apoptosis level to oxaliplatin by TUNEL level *in vivo*. **G**. Down-regulation of miR-503-5p significantly slowed down CRC-OxR cells growth under oxaliplatin treatment by Ki67 level, and increased CRC-OxR cells apoptosis level to oxaliplatin by TUNEL level *in vivo*. The images are representative of multiple fields of tumor sections from each group. Percentage of positive Ki67 and TUNEL staining cells were determined as described in *Materials and Methods*. The data are presented as the mean ± SD. *p < 0.05, **p<0.01, *** *p* < 0.001.

The above results showed that overexpression of miR-503-5p induced oxaliplatin resistance in CRC cells *in vitro*. To verity this results *in vivo*, a CRC xenograft model was established by subcutaneously inoculating nude mice, injecting with HCT116 cells (2 × 10^6^) expressing ctrl vector or miR-503-5p, or HCT116-OxR cells (2 × 10^6^) expressing ctrl vector or miR-503-5p miArresst. After 2 weeks, we divided those mice randomly into two groups. 10 mg/kg oxaliplatin was injected administered by i.p. into one group, and control solution was treated to the other group every 4 days. To determine the therapeutic sensitivity during the treatment, we monitored the tumor volume of each group. Xenograft tumor growth curves demonstrated that tumors had a independent similar rate after miR-503-5p expressed. Moreover, a big difference was showed in the response of tumors under oxaliplatin treated. Tumors contained with control vector (control tumors) growing was inhibited during oxaliplatin treated, in addition tumors contained with miR-503-5p (miR-503-5p tumors) kept on growing at a stable rate (Figure 4A). Furthermore, the size (Figure 4C) and weight (Figure 4D) of miR-503-5p tumors manifested obviously smaller reduction of after oxaliplatin treatment than control tumors. As a complementary approach in the group of OxR-tumors, we observed that OxR-tumors with miR-503-5p miArresst expressing cells (miR-503-5pi OxR-tumor) exhibited increased effectiveness of oxaliplatin in the inhibition of tumor growth compared to the OxR-tumors with control vector-expressing cells (control OxR-tumor) (Figure 4A), the same effect on tumor size (Figure 4C), and tumor weight (Figure 4E). Next, We used TUNEL assay to determine the tumors apoptotic index, Ki67 staining was used to determine the proliferative levels of tumors (Figure 4F & 4G). The results showed that miR-503-5p overexpressed significantly decreased the growth-inhibitory effect of oxaliplatin in CRC-Par cells by Ki67 level, and decreased oxaliplatin-induced apoptosis of CRC-Par cells. Down-regulation of miR-503-5p suppressed CRC-OxR cell growth under oxaliplatin treatment, and increased oxaliplatin-induced apoptosis of CRC-OxR cells. The above data indicated that miR-503-5p could modulate the oxaliplatin effectiveness to CRC *in vivo*. Taken together, the results of *in vitro* and *in vivo* suggest miR-503-5p could play an crucial role in drug resistance of CRC cells.

### p53 suppresses miR-503-5p expression in CRC cells

**Figure 5:**
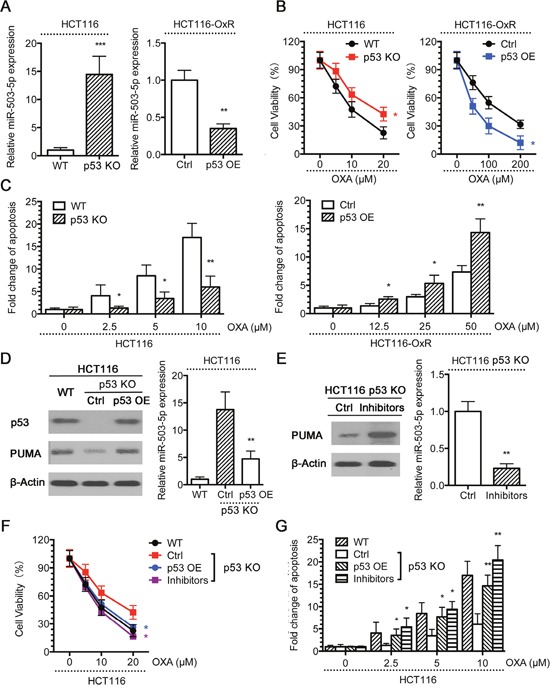
p53 suppresses miR-503-5p expression **A**. miR-503-5p expression was higher in p53 knock out (KO) than wild type (WT) HCT116 cells, and decreased in the p53 overexpression (OE) HCT116-OxR cells compared to control plasmid, by qPCR assays. WT and p53 KO HCT116 cells, Ctrl and p53 OE HCT116-OxR cells were treated with different concentrations of OXA for 48 hrs. **B**. MTT assays and **C**. DNA fragmentation assays showed that HCT116 p53 KO cells were more resistant to OXA-induced apoptosis than WT cells, and p53 OE HCT116-OxR cells were more sensitive to OXA-induced apoptosis than Ctrl cells. **D**. p53 was ectopically expressed in HCT116 p53 KO cells. Western blot analysis showed restored expression of p53 and PUMA expression (left panel), qPCR assays showed that restored p53 expression suppressed miR-503-5p expression (right panel).**E**. The miR-503-5p inhibitor was transfected into HCT116 p53 KO cells. As a result, miR-503-5p expression was suppressed as determined by qPCR assays (left panel) and PUMA expression was increased as examined by western blot assays (right panel). Cells were treated with different concentrations of OXA for 48 hrs. **F**. MTT assays and **G**. DNA fragmentation assays showed that restored expression of p53 and miR-503-5p inhibitor re-sensitized p53 KO cells to OXA-induced apoptosis. The data are presented as the mean ± SD of triplicate experiments. *p < 0.05, **p<0.01, *** *p* < 0.001.

Our above data suggested that miR-503-5p promotes drug resistance CRC cells. Thus, we wanted to determine the mechanisms regulating miR-503-5p expression. Previous studies have indicated that p53 contributes to oxaliplatin and other chemotherapeutic drug-induced apoptosis, thus playing a central role in oxaliplatin resistance [35, 36]. Since our results have shown that the PUMA expression, which is up-regulated by p53, is decreased in oxaliplatin resistant CRC cells, and the miR-503-5p expression is increased, we hypothesized that the expression of miR-503-5p be suppressed by p53 in CRC cells. To confirm this hypothesis, we compared the expression of miR-503-5p between HCT116 wild type (WT) and p53 knock out (p53 KO) cells, and in HCT116-OxR control (Ctrl) and p53 overexpressing (p53 OE) cells. miR-503-5p expression was decreased in p53 KO cells compared to WT cells, and decreased in p53 OE compared to Ctrl cells (Figure 5A). And, p53 KO cells showed more resistance to oxaliplatin than WT cells, and overexpression of p53 could re-sensitize HCT116-OxR cells to oxaliplatin (Figure 5B). The same results were obtained in apoptosis assays (Figure 5C). Restored p53 expression in p53 KO cells decreased miR-503-5p expression (Figure 5D, right panel), showing that p53 inhibits the expression of miR-503-5p. It is worth noting that, PUMA expression was increased in p53 re-expressed cells (Figure 5D, left panel). Therefore, p53 expression restoration of HCT116 p53 KO cells sensitized the cells to Oxaliplatin treatment (Figure 5F & 5G). To confirm whether miR-503-5p is liable for the resistance of p53 KO cells to oxaliplatin treatment, the miR-503-5p inhibitors were transfected into p53 KO cells. The inhibitors decreased miR-503-5p expression in p53 KO cells as documented by RealtimePCR (Figure 5E, right panel), which led to increasing expression of PUMA (Figure 5E, left panel). Reduction of miR-503-5p effectively sensitized oxaliplatin to p53 KO cells, as evidenced by a left shift of the growth inhibition curve (Figure 5F), and increased apoptosis (Figure 5G). These examines indicate that inhibition of miR-503-5p expression is one of the mechanisms by p53 promotes oxaliplatin-induced apoptosis, furthermore the raising miR-503-5p expression contributes to oxaliplatin resistance in p53 KO cells.

### Negative correlation of miR-503-5p and PUMA in human CRC tissues

**Figure 6:**
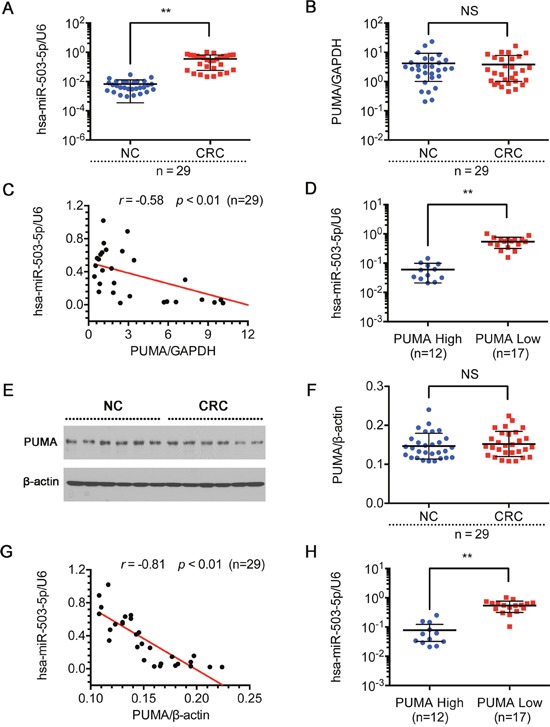
Expression of miR-503-5p and PUMA in human CRC tissue sample Relative expression levels of **A**. miR-503-5p and **B**. PUMA mRNA were detected in non-tumorous colon (NC) tissues and CRC tissues (n=29) via qRT-PCR. Abundance of miR-503-5p and PUMA was normalized to U6 RNA and GAPDH, respectively. **C**. Expression levels of miR-503-5p and PUMA mRNA are inversely correlated among all the tissue samples (n = 29) as indicated by two-tailed Pearson's correlation analysis, *r* = −0.58; *p* < 0.01. **D**. miR-503-5p expression levels were inversely correlated with PUMA mRNA expression in CRC tissues. **E** & **F**. PUMA protein level were detected in normal colon tissues (NC) and CRC tissues (n=29) via Western blot analysis. Abundance PUMA was normalized to β-actin. **G**. Expression levels of miR-503-5p and PUMA protein level are inversely correlated among all the tissue samples (n = 29) as indicated by two-tailed Pearson's correlation analysis, *r* = −0.81; *p* < 0.01. **H**. miR-503-5p expression levels were inversely correlated with PUMA protein level expression in CRC tissues. The data are presented as the mean ± SD. *p < 0.05, **p<0.01, *** *p* < 0.001.

To better understand the potential clinicopathological implications of miR-503-5p, we analyzed the expression of miR-503-5p and PUMA in 29 pairs of tissues samples (non-tumorous colon (NC) tissues and CRC tissues) by qPCR. Table 2 manifest the correlation between expression of miR-503-5p and clinicopathological characteristics of CRC. And there was a statistically significant association between miR-503-5p expression and degree of differentiation in this study. The median expression of miR-503-5p in poorly differentiated tissues was higher than in the well and moderately differentiated tissues (*p* < 0.01, Mann-Whitney test). In addition, miR-503-5p expression was reduced in CRC tissues compared with the corresponding non-tumorous colon (NC) samples (Figure 6A). To investigate the association between miR-503-5p and PUMA, we measured the PUMA expression in tissues by qPCR and western blotting. However, we did not find any significant differences in PUMA mRNA levels (Figure 6B) or protein levels (Figure 6E & 6F) between CRC and NC tissues. In Figure 6C and 6G, each point in the scatter graph represents an individual sample with the relative miR-503-5p level indicated on the y-axis, and the PUMA expression indicated on the x-axis. The correlation coefficient indicated that there is a strong negative relationship between miR-503-5p and PUMA mRNA expression (*r* = -0.58, *p* < 0.01) (Figure 6C), or PUMA protein expression (*r* = -0.81, *p* < 0.01) (Figure 6G) in CRCs. The Spearman's rank statistical test was used for analysis. The expression of miR-503-5p and PUMA mRNA were described by the formulas 2^−ΔCt^ and 2^−^, and the levels of PUMA protein was described by the detected bands’ intensity of PUMA protein/β-actin protein. We found that high miR-503-5p expression was always associated with low PUMA expression. Using the Mann-Whitney test, we have shown that miR-503-5p expression inversely correlates with PUMA expression (P<0.01) (Figure 6D & 6H).

**Table 2:** The relationship between clinicopathological parameters and miR-503-5p expression in human coloretal carcinoma

Variable	Number of cases	%	Median expression of miR-503-5p/U6	*p*-value
***Age (years)***				
≥ 60	16	55%	0.3519	0.4734
< 60	13	45%	0.3473	
***Gender***				
Male	12	41%	0.3443	0.4342
Female	17	59%	0.3537	
***Degree of differentiation***				
Well and moderately differentiated	14	48%	0.2613	< 0.01
Poorly differentiated	15	52%	0.4325	
***Lymph node status***				
Metastasis	16	55%	0.3732	0.2176
No metastasis	13	45%	0.3211	
***Extent of invasion***				
Invasion	17	59%	0.3628	0.3287
No invasion	12	41%	0.3315	
***TNM stage***				
Stage I/II	12	41%	0.3357	0.4142
Stage III/IV	17	59%	0.3592	

## DISCUSSION

Previous works have suggested that miRNAs play an crucial role in cancer progression, and that miRNA expression signatures may be used as potential diagnostic and prognostic markers for cancer diagnosis and treatment [18–25]. However, the specific role of miRNAs in development of cancer MDR remains largely unexplored. Our research demonstrates participation of miRNAs in the development of MDR in CRC cells, indicating that miRNAs may act as potential targets for chemo-sensitizing strategies.

We have found that ten microRNAs exhibit differential expression between two parental CRC cell lines HCT116 and HT29, and their oxaliplatin-resistant derivative cells (Table 1), suggesting that them could play paramount role in the oxaliplatin resistance in CRC. Nowadays, the major clinical treatment strategy of colorectal carcinomas is platinum drugs. Thence the platinum-drug resistance turns into a urgent clinical problem that needs to be resolved. It is critical to delineate the drug resistance mechanisms to commonly used therapeutic agents, such as oxaliplatin. Importantly, the mechanisms underlying the resistance to oxaliplatin have not been completely clarified. Our works built a link between miR-503-5p and oxaliplatin resistance. This link likely relates a PUMA-mediated mechanism. In the first place, by miRNA microarray profiling, we showed that miR-503-5p is expressed at elevated levels in two oxaliplatin-resistant cell lines. And then, we demonstrated that miR-503-5p regulates PUMA expression by directly binding to the PUMA 3′UTR site. Third, we demonstrated that overexpression of miR-503-5p protects parental CRC cells from oxaliplatin-induced apoptosis, and that suppression of miR-503-5p sensitizes resistant CRC cells to oxaliplatin *in vitro* and *in vivo*. Finally, we have defined a novel p53/miR-503-5p/PUMA signaling axis that mediates the response of CRC cells to oxaliplatin. In wild type p53 cells, miR-503-5p expression was suppressed and oxaliplatin treatment induced apoptosis, while in p53 KO cells, miR-503-5p expression was up-regulated and cells became resistant to oxaliplatin. The miR-503-5p inhibitors could re-sensitize p53 KO cells to oxaliplatin treatment.

Previous study determined that p53 regulate PUMA in transcriptional level. Here in our results demonstrated that PUMA is also a direct target of miR-503-5p in CRC cells. Since p53 suppresses expression of miR-503-5p, p53 could regulate PUMA expression in two different mechanisms: regulating its promoter activity through transcriptional factors and its 3′UTR through miR-503-5p. Since resistance to oxaliplatin treatment is one of the main reason for the failure of chemotherapy in CRC, the findings of miR-503-5p as a contributing factor of MDR and the certification of the p53/miR-503-5p/PUMA signaling axis should help design strategies to increase efficacy of oxaliplatin treatment of CRC.

Interestingly, we found that miR-503-5p was up-regulated in CRC tissues compared to NC tissues, and a strong negative relationship was observed between miR-503-5p and PUMA gene and protein expression in CRC tissues. Our results present the first evidence that miRNAs may be involved in the development of MDR in colorectal carcinoma cells by P53/PUMA pathway. In addition, our results demonstrate that miR-503-5p may modulate PUMA-mediated MDR of tumor cells to chemotherapy drugs. Our data manifestedthat the overexpression of miR-503-5p contributed to the loss of PUMA and oxaliplatin resistance in CRC-OxR cells, suggesting that targeting P53/miR-503-5p/PUMA might have significance for prevention and reversion of MDR in CRC.

In conclusion, we have demonstrated that miR-503-5p expression negatively correlates with PUMA expression in colorectal carcinoma cells. Moreover, our findings of miR-503-5p as a regulatory of drug response in CRC offers another potential therapeutic way. Reduction of miR-503-5p expression may increase chemosensitivity in CRC treatment. Our findings contribute to the understanding of MDR regulation in cancer cells. Additionally, these findings may be beneficial for future research of predicting drug resistance in patients, and designing personalized therapy for colorectal carcinoma patients.

## MATERIALS AND METHODS

### Cell lines and reagents

Human colorectal carcinoma cell lines HCT116 and HT29 (obtained from the Cell Bank of Chinese Academy of Science) and their oxaliplatin-resistant variants HCT116-OxR and HT29-OxR (established and maintained in our laboratory) were maintained in RPMI 1640 medium (Gibco Industries, Inc.) with 10% fetal bovine serum (Gibco Industries, Inc.) at 37°C in a humidified atmosphere with 5% CO_2_. HCT116-OxR and HT29-OxR cells were seeded in a medium containing 5 μg/ml of oxaliplatin (OXA) to maintain their drug-resistance phenotype.

MiR-503-5p mimics, negative control mimics, miR-503-5p inhibitors, and negative control inhibitors were purchased from Exiqon Inc. (Woburn, MA). cDNA encoding miR-503-5p precursor and miArrest were cloned into pMCS-CMV lentiviral vector purchased from GeneChem, Shanghai, P.R. China. PUMA knock down shRNA plasmid and p53 CRISPR/Cas9 KO plasmid were purchased from Santa Cruz Biotechnology (Santa Cruz, CA). PUMA overexpression plasmid and p53 overexpression plasmid were purchased from Addgene (Cambridge, MA). 293T packaging cells were co-transfected with pPackH1 packaging plasmid mix (SBI, Mountain View, CA) and the lentiviral vectors using Fugene HD (Promega, Madison, WI). Viruses were harvested 48 hours later and used to infect target cells.

### Tissue samples

Human CRC and their corresponding NC (non-tumorous colon) samples were collected at the time of surgical resection at Putuo Hospital, Shanghai University of Traditional Chinese Medicine, P.R. China, from January 2010 to December 2011. Written informed consent was obtained from the patients, in accordance with the institutional guidelines, before sample collection, and the study was approved by the Committees for the Ethical Review of Research at the Putuo Hospital, Shanghai University of Traditional Chinese Medicine, P.R. China. The methods were performed in accordance with the approved guidelines. All patients had a histological diagnosis of colorectal cancer and received radical resection. None of the patients included in the study had received neoadjuvant therapy before surgery. Samples were immediately snap frozen in liquid nitrogen and stored at −80°C.

### miRNA microarray analysis

Prior to experimentation, HCT116-OxR cells and HT29-OxR cells were cultured 1 week without oxaliplatin. Total RNA from HCT116, HCT116-OxR, HT29 and HT29-OxR cell lines was isolated with Trizol reagent (Invitrogen, Carlsbad, CA) and miRNA fraction was further purified using a mirVana™ miRNA isolation kit (Ambion, Austin, TX). The isolated miRNAs was labeled with Hy3 using the miRCURY™ Array Labelling kit (Exiqon, Vedbaek, Denmark) and hybridized on a miRCURYTM LNA microRNA Array (v 8.0, Exiqon) as described [37]. Microarray images were acquired using a Genepix 4000B scanner (Axon Instruments, Union City, CA) and processed and analyzed with Genepix Pro 6.0 software (Axon Instruments) and Excel.

### Quantitative RT-PCR

To prepare total RNA from tissues, the frozen tissues were ground into finely ground particles after 5-mm^3^ sections of each sample were cut, and then the tissue particles were subjected to extraction of RNA with TRIzol (Invitrogen). Total RNA was extracted from cultured HCT116, HCT116-OxR, HT29 and HT29-OxR cells with Trizol (Invitrogen). The concentration of total RNA was quantitated by measuring the absorbance at 260 nm. Expression of mature miRNAs was assayed using stem-loop RT followed by real-time PCR analysis as previously described [38]. All reagents for stem-loop RT were obtained from Applied Biosystems (Foster City, CA). The relative amount of each miRNA was normalized to U6 snRNA. The relative expression levels of each cell line of each group were measured using the 2^−ΔΔCt^ method [38]. Briefly, the average ΔCt of each group was calculated by following formula: ΔCt = average miR-503-5p Ct - average of HK (housekeeping) gene (U6 snRNA)' Ct. ΔΔCt was calculated by ΔΔCt = ΔCt of control group- ΔCt of the treated group. The fold change for miR-503-5p expression level in cell lines was calculated using 2^−ΔΔCt^. The results are presented as fold change for each miRNA from HCT116-OxR or HT29-OxR cells relative to its control (HCT116 or HT29 cells). And the miR-503-5p expression level in tissues was calculated using 2^−ΔCt^. The primers of miR-503-5p and U6 snRNA used for stem-loop RT-PCR were purchased from QIAGEN Inc. (Valencia, CA). For SYBR Green quantitative PCR amplifications, reaction was performed in a 20 ml reaction volume contained SYBR Green PCR Master Mix (Applied Biosystems). The relative expression levels of each cell line of each group were measured using 2^−ΔΔCt^ methods as before. And the relative expression level of tissues was measured using the 2^−ΔCt^. The primer sequences as followed: PUMA, 5′- GCGAGACTGTGGCCTTGTGT-3′, 5′-CGTTCCAGGGTCCACAAAGT-3′; GAPDH, 5′-CTCCATCCTGGCCTCGCTGT-3′, 5′-GCTGTCAC CTTCACCGTTCC-3′.

### Luciferase activity assay

The wild-type and mutated miR-503-5p putative targets on PUMA 3′UTR were cloned into pGL3-promoter vector. The cells (2 ×10^4^) were cotransfected with 500 ng of pGL3-PUMA-WT or pGL3-PUMA-Mut constructs with miR-503-5p mimics. Each sample was cotransfected with 50 ng of pRL-SV40 plasmid expressing renilla luciferase to monitor the transfection efficiency. A luciferase activity assay was performed 48 h after transfection with the dual luciferase reporter assay system (Promega, Wisconsin, USA). The relative luciferase activity was normalized to the renilla luciferase activity.

### Western blot analysis

Proteins were resolved on SDS/PAGE gel and subjected to immunoblot analysis using monoclonal antibodies against PUMA, cleaved PARP, p53 or β-actin (Cell Signaling Technology, US). All antibodies were used at 1 μg/ml of working concentration in PBS with 5% dried-milk. The membrane was further probed with horseradish peroxidase (HRP)-conjugated rabbit anti-mouse IgG (Santa Cruz, 1:2000) and the protein bands were visualized using enhanced chemiluminescence (Amersham Pharmacia Corp, Piscataway, NJ). Quantification of protein bands was performed using the ImageJ software.

### Cell viability and apoptosis assays

Colon cancer cells were plated in 96-well plates and treated with oxaliplatin for indicated times. After 48 h, cell viability was assessed using an MTT assay (Sigma, St. Louis, MO). The absorbance at 490 nm of each well was read on a spectrophotometer (Bio-Rad, Hercules, CA.) Cell viability was calculated as a ratio of OD values of drug treated samples to those of controls. Apoptosis was detected using a DNA fragmentation ELISA kit (Roche, Indianapolis, IN).

### Caspase-3 activity assay

HCT116-OxR cells (1 × 10^6^/dish) were seeded in 10 cm dishes in antibiotic-free medium and transfected with control oligonucleotide (40 nM) or inhibitors (40 nM) using Lipofectamine 2000 (Invitrogen). After 48h, cells were collected, washed three times with PBS and re-suspended in 50 mM Tris–HCl (pH 7.4), 1 mM EDTA, and 10 mM ethylene glycol tetraacetic acid (EGTA). Cell lysates were clarified by centrifugation at 18,000g for 3 min, and clear lysates containing 50 mg of protein were incubated with 100 mM of enzyme-specific colorimetric substrate at 37°C for 1h. The activity of caspase-3 was expressed as the cleavage of colorimetric substrate by measuring absorbance at 405 nm.

### *In vivo* xenograft model

Experiments involving animals were approved by Institutional Review Board of the Putuo District Center Hospital. HCT116 cells (2×10^6^) expressing miR-503-5p or an control vector were injected into the flank of male athymic nude mice (4-5 weeks old); and HCT116-OxR cells (2×10^6^) expressing miR-503-5p miArresst (miR-503-5pi) or an control vector were injected into the flank of male athymic nude mice (4-5 weeks old). Two weeks after injection, oxaliplatin (10 mg/kg) or carrier was administered by i.p. injection every 4 days for 4 weeks [23]. Tumor volumes (V) were calculated by the formula V = W^2^ × L × 0.5, where W represents the largest tumor diameter in centimeters and L represents the next largest tumor diameter. The relative tumor volumes (RTV) were calculated by RTV = Vx/V_0_ where Vx is the volume in cubic millimeters at a given time and V_0_ is the volume at the beginning of the treatment [39]. Tumors were dissected out and weighted.

All animal experiments were approved and supervised by the institutional animal care and use committee of Putuo Hospital, Shanghai University of Traditional Chinese Medicine, P.R. China. All animal studies were conducted in accordance with the National Institute of Health guidelines for the Care and Use of Laboratory Animals.

### TUNEL and Ki67 staining

Formalin fixed paraffin embedded (FFPE) tissue blocks of tumors were stained for TUNEL and Ki67 using the procedure described previously [40]. Three tumors from each group were analyzed. Ten histologically similar fields were randomly selected from each slide for analysis. Apoptosis and proliferation of tumor cells were quantified by counting the cells and calculating the percentage of positively stained cells for TUNEL and Ki67, respectively.

### Statistical analysis

Each experimental value was expressed as means ± standard deviation (SD). Statistical analysis was performed using the T-test to evaluate the significance of differences between cell lines groups considered as *p < 0.05; **p < 0.01. All data points represented the mean of triplicates. Statistical analysis of tissues samples was performed using the Mann–Whitney test to evaluate the significance of differences between groups.
